# Spiking neural network model of cortical auditory source segregation

**DOI:** 10.1186/1471-2202-15-S1-P50

**Published:** 2014-07-21

**Authors:** Lakshmi Krishnan, Michael Campos, Shihab Shamma

**Affiliations:** 1 Department of Electrical and Computer Engineering, University of Maryland, College Park, MD 20783, USA; 2 Qualcomm Research, San Diego, CA 92121, USA; 3 Department Etude Cognitive, Ecole Normale Suprieure, Paris 75005, France

## 

Humans have the remarkable ability to tune into a particular voice even in loud noisy environments. The neural underpinnings of this amazing perceptual phenomenon are not yet fully understood. Recent EcoG [1] and MEG studies [2] have established that the neural representation of the attended speaker’s speech is much stronger than the unattended (distractor) speech when human subjects are asked to pay attention to a target speaker in a mixture of speech. How the brain sieves through the mixture waveform to enhance the target speaker’s speech and attenuate the background acoustic scene is still being investigated. In this work, we propose a spiking neural network architecture based on the theory of temporal coherence [3] to achieve auditory source segregation.

Our model does not require training on the background noise or prior exposure to the target speech. Along with using bottom-up spectro-temporal features and pitch features, the model can also accommodate top-down attentional mechanisms to generate segregated phase locked neural representations to target speaker’s speech envelope. The model comprises of a feature extraction stage followed by clustering stage. The feature extraction stage mimics the auditory pathway starting from a cochlear representation followed by a multi-resolution analysis of the cochlear output using a bank of band-pass filters (cortical stage), to provide a rich timbre representation. Dominant pitch tracks are extracted from the sound mixture and processed through the same set of band-pass filters as the timbre channels. The output of the feature extraction stage comprising of the pitch and timbre channels are transduced into a spike-based representation using leaky integrate and fire neurons with time constants tuned to the bandwidth of the multi-resolution band-pass filters. The clustering stage comprises of a bank of coincidence detector neurons. Using the pitch signals as anchors the coincidence detector neurons can segregate the two sources from the mixture timbre representation. Thus, the output of the coincidence detector neurons comprises only of responses phase locked to the envelope of a single source.

This model does not require any weight learning, is unsupervised and can segregate sources online. Previous studies on correlation based sound segregation employed network of neurons with intrinsic oscillator dynamics [4]. In this work, clustering of features belonging to a single source is driven only by the temporal coherence of spectro-temporal features of the given source. This spike-based representation provides an easy mechanism to group coherent features, which otherwise would require computationally expensive numerical routines for online, adaptive principal components analysis. Future work is aimed at reconstructing the speech waveform from the segregated spike trains.
